# Heart Failure Mortality Before and After COVID-19: Demographic Variations and Urbanization Patterns in the United States (2018-2023)

**DOI:** 10.7759/cureus.117568

**Published:** 2026-10-06

**Authors:** Andrew López Sánchez, Lissett Ferrer Orozco, Elmer D Perez Valdivia, Francisco J Santana Mercedes, Aitor J Roche Perez, Carmen Soler Martin, Gabriela Montano Diaz, Yanisleidy Valladares Urquiza, Javier Peña Angel, Dailyn Rivera Hernández, Mailyn Marrero Herrera, Carlos Acosta-Batista

**Affiliations:** 1 Internal Medicine, Primary Care - Research Initiative (PCRI), Miami Lakes, USA; 2 Cardiology, Primary Care - Research Initiative (PCRI), Miami Lakes, USA; 3 Intensive Care Unit, Primary Care - Research Initiative (PCRI), Miami Lakes, USA; 4 Geriatrics, Primary Care - Research Initiative (PCRI), Miami Lakes, USA; 5 Internal Medicine, Iditalya 10 Medical Center, Tampa, USA; 6 Primary Care, Primary Care - Research Initiative (PCRI), Miami Lakes, USA; 7 Internal Medicine and Hematology, Primary Care - Research Initiative (PCRI), Miami Lakes, USA; 8 Radiology, Primary Care - Research Initiative (PCRI), Miami Lakes, USA; 9 Psychiatry, Larkin Community Hospital, Miami, USA; 10 Clinical Research, Primary Care - Research Initiative (PCRI), Miami Lakes, USA; 11 Research and Development, Tricocenter Miami, Miami Lakes, USA

**Keywords:** asian health, cdc wonder, health disparities, heart failure, hispanic health, joinpoint regression, mortality trends

## Abstract

Background: Contemporary evidence on heart failure (HF) recorded as the underlying cause of death remains fragmented across demographic groups and levels of urbanization. The 2018-2023 interval includes two years before the emergence of COVID-19 (2018-2019) and four years after its emergence (2020-2023).

Objective: We aim to characterize the national underlying cause of HF mortality among US adults aged 45 years or older from 2018 through 2023; describe differences by sex, age, race, and Hispanic origin; and quantify changes in absolute death counts across the six National Center for Health Statistics (NCHS) urbanization categories.

Hypothesis: We hypothesized that the national age-adjusted HF mortality rate would increase and that demographic differences and variation in geographic death counts would persist.

Methods: This retrospective, population-based, serial cross-sectional study used Centers for Disease Control and Prevention Wide-ranging Online Data for Epidemiologic Research (CDC WONDER) Underlying Cause of Death data (International Classification of Diseases, 10th Revision (ICD-10) I50). Age-adjusted mortality rates (AAMRs) per 100,000 were evaluated overall and across demographic groups; age-specific crude rates were used for age groups. Zero-joinpoint log-linear models estimated annual percent change (APC) for the overall population, race categories, and Hispanic-origin categories. Race and Hispanic origin were analyzed separately and may overlap. Urbanization analyses used death counts and percentage changes; urbanization-specific population-adjusted rates were not estimated. Deaths listing HF only as a contributing cause were outside the case definition.

Results: There were 512,960 deaths with HF as the underlying cause; the aggregate AAMR was 59.6 per 100,000. No statistically significant overall temporal trend was detected (APC: 0.59%; 95% confidence interval (CI): -0.37 to 1.57; p = 0.227). Men had a higher aggregate AAMR than women (68.1 versus 53.0), and Black adults had the highest racial AAMR (70.9). Asian adults had a lower aggregate AAMR than White and Black adults (22.9); Hispanic adults had a lower AAMR than non-Hispanic adults (35.2 versus 61.9). In their respective analyses, Asian and Hispanic groups had increasing trends at the nominal 0.05 significance level (APC: 1.30%, p = 0.047, and 1.96%, p = 0.030, respectively). Age-specific mortality increased sharply with age. Death counts increased across all six urbanization categories, most in Medium Metro counties (+11.0%).

Conclusions: No statistically significant national trend was detected during 2018-2023, while descriptive demographic differences and nominally significant increases in the Asian race and Hispanic-origin analyses were observed. These separate analyses do not establish differences between subgroup slopes or narrowing mortality gaps. Geographic findings describe absolute burden, not population-adjusted risk. The results concern HF as the underlying cause and do not measure total HF-associated mortality.

## Introduction

Heart failure (HF) represents a major and growing public health burden in the United States because of its high prevalence, mortality, recurrent hospitalization, and healthcare utilization. Approximately 7.7 million US adults were living with HF in 2021-2023, and 89,795 deaths were attributable to HF in 2023 [1]. This burden is expected to increase with population aging and the rising prevalence of cardiometabolic risk factors; HF prevalence is projected to increase from 2.7% in 2020 to 3.8% by 2050 [2].

Although HF-related mortality declined during the early 2000s, this favorable trend slowed and subsequently reversed. National analyses using Centers for Disease Control and Prevention Wide-ranging Online Data for Epidemiologic Research (CDC WONDER) have documented increasing HF-related mortality after approximately 2011-2012, including among older and middle-aged adults, with additional perturbation during the COVID-19 era [3-8]. These observations make the 2018-2023 interval epidemiologically informative because it includes 2018-2019 before the emergence of COVID-19 and 2020-2023 after its emergence. These calendar-year descriptions do not define mutually exclusive pandemic phases or constitute a formal pre/post comparison.

The mortality burden has not been distributed uniformly. Prior studies have documented differences by sex, race and ethnicity, geographic region, and urbanization, with higher population-adjusted HF mortality reported among men, non-Hispanic Black adults, and many rural populations [3,4,6-9]. Some prior studies have reported lower aggregate mortality in selected Hispanic-origin and Asian race comparisons. A Florida ecological analysis reported an inverse association between Hispanic population concentration and cardiovascular mortality [10]; that area-level finding does not establish individual protection or explain national HF-specific mortality. Similarly, Siddiqi et al. demonstrated that Asian American adults consistently maintained the lowest heart failure-related mortality rates among older US adults prior to the pandemic [5]. Contemporary underlying-cause HF mortality trends among adults aged 45 years or older in these population groups merit further description. Race and Hispanic origin are separate classifications, so these groups may overlap and should not be treated as mutually exclusive cohorts. Potential explanations include differences in cardiovascular risk-factor burden, socioeconomic conditions, continuity of care, medication access, specialist availability, and other social determinants of health [9,11,12]. Because these factors are not measured in aggregate mortality files, however, they should be used to contextualize rather than causally explain observed subgroup differences.

Contemporary evidence nevertheless remains fragmented. Several national analyses ended in 2018-2021 or focused on selected age groups [3,5,8], whereas more recent studies extending through 2022-2023 examined middle-aged adults or place of death [4,13]. In addition, many prior studies combined HF listed as an underlying or contributing cause of death, an approach that captures a broader but conceptually different mortality burden. Underlying-cause HF mortality during 2018-2023 therefore remains incompletely characterized across multiple demographic dimensions and the full National Center for Health Statistics (NCHS) urban-rural continuum within a single national analysis.

The primary objective of this study was to characterize national trends in mortality attributable to HF as the underlying cause of death among US adults aged 45 years or older from 2018 through 2023. Secondary objectives were to quantify differences by sex, age, race, Hispanic origin, and race-by-sex strata; to evaluate prespecified temporal trends using joinpoint regression; and to describe changes in the absolute number of HF deaths across the six NCHS urbanization categories as a description of geographic burden rather than population-adjusted mortality risk. We hypothesized that the national age-adjusted mortality rate (AAMR) would increase over the study period and that demographic rate differences and variation in geographic death counts would persist.

## Materials and methods

Study design and period

This retrospective, population-based, serial cross-sectional trend study examined mortality attributable to HF among US adults aged 45 years and older for a prespecified analytic period from 2018 through 2023. The study is ecological and descriptive; associations observed across population strata were not interpreted as individual-level causal effects.

Data source

Mortality data were obtained from the Centers for Disease Control and Prevention Wide-ranging Online Data for Epidemiologic Research (CDC WONDER) Underlying Cause of Death, 2018-2024, Single Race database [14]. The database compiles de-identified death-certificate data for residents of all 50 US states and the District of Columbia. Cause of death is coded according to the International Classification of Diseases, 10th Revision (ICD-10) [15].

Study population and inclusion/exclusion criteria

The study population comprised US resident decedents aged 45 years or older. Deaths were included if the death occurred between January 1, 2018, and December 31, 2023, inclusive, and the death certificate listed an ICD-10 code within the I50 category as the underlying cause of death. Decedents younger than 45 years, deaths of non-US residents, and deaths outside the 50 states and District of Columbia were excluded. Records with unknown demographic values were excluded from stratum-specific rate comparisons when a valid population denominator was unavailable.

Data collection and variable definitions

The primary outcome was a death for which ICD-10 category I50 was selected as the underlying cause. This is a cause-specific death-certificate endpoint, not all-cause mortality among people with HF and not all deaths involving HF. Deaths in which HF appeared only as a contributing cause were not included; thus, this analysis captures a subset of the broader HF-associated mortality burden. The complete I50 category in the CDC WONDER Underlying Cause of Death database includes I50.0 (congestive heart failure), I50.1 (left ventricular failure), and I50.9 (heart failure, unspecified). For most stratified extractions, cases were identified using CDC WONDER's predefined "Heart failure (I50)" category. A separate query selecting I50.0, I50.1, and I50.9 individually was used to characterize the distribution of HF subtype coding.

Age was grouped as 45-54, 55-64, 65-74, 75-84, and 85 years or older. Sex was categorized as female or male as recorded on the death certificate. Race was categorized using the CDC WONDER Single-Race six-category classification: White; Black or African American; American Indian or Alaska Native; Asian; Native Hawaiian or Other Pacific Islander; and more than one race. Hispanic origin was analyzed separately as Hispanic or Latino, Not Hispanic or Latino, or Not Stated. Each race category includes persons of any Hispanic origin, and each Hispanic-origin category includes persons of any race. Race and Hispanic origin therefore constitute separate, potentially overlapping analytic groupings, not mutually exclusive cohorts. Race and sex were additionally cross-classified to describe AAMRs across race-by-sex strata. These cross-classified comparisons were descriptive and were not treated as a formal statistical interaction test.

County of residence was classified using the 2013 NCHS Urban-Rural Classification Scheme for Counties: Large Central Metro, Large Fringe Metro, Medium Metro, Small Metro, Micropolitan, and Noncore [16]. All six categories were retained to describe the distribution of absolute HF mortality burden across the full urban-rural continuum.

The selected Single-Race queries did not supply the denominators needed for the urbanization analyses. WONDER documents limitations for county/urbanization rates with 10-year age groups and for its standard county-level age-adjusted rates; five-year-age population queries can be available in other configurations. In addition, national urbanization queries including Connecticut have denominator limitations from 2022 onward [14]. The restriction here is specific to the query structure and period, rather than universal suppression of all geographic populations. Accordingly, the urbanization analysis in this study was restricted to absolute death counts and percentage change, calculated as 100 × (deaths in 2023 - deaths in 2018) / deaths in 2018. An external-denominator analysis would require harmonized age groups, population-estimate vintages, and county boundaries; no such alternative rate analysis was performed. No population-adjusted urbanization-specific mortality rates were estimated, and geographic findings were not interpreted as differences in mortality risk or access to care.

Data were extracted directly from the CDC WONDER online query system using the Underlying Cause of Death, 2018-2024, Single Race database, with all results filtered to 2018-2023. The cause-of-death filter included I50.0, I50.1, and I50.9. Age was restricted to 45 years or older. Separate queries were performed for the overall annual trend, sex, race, Hispanic origin, race-by-sex strata, age group, and the 2013 six-level urbanization classification, as well as selected combinations such as year by race and year by Hispanic origin.

Raw exports preserved CDC WONDER output designations including Suppressed, Unreliable, Not Applicable, and Not Available; these values were not replaced with zeros. Data extraction was performed independently by two analysts between August 10 and 12, 2026, using identical query parameters. The two extractions were compared only after completion, and discrepancies were resolved by re-querying CDC WONDER directly. A reconciled master dataset and data dictionary were then compiled.

The US 2000 standard population was used for age adjustment. CDC WONDER-provided AAMRs were used for eligible overall and demographic groupings. Age-group results were reported as age-specific crude rates rather than AAMRs. For urbanization, only death counts and changes in counts were analyzed because the required denominators for age-adjusted rates were unavailable in the selected query structure.

The principal mortality measure was the age-adjusted mortality rate (AAMR) per 100,000 population, standardized to the US 2000 standard population. Age-specific crude mortality rates per 100,000 were used for the predefined age groups. Ninety-five percent confidence intervals (CI) for age-adjusted rates followed CDC/NCHS methods, including the Fay-Feuer gamma approximation where applicable [17]. Rate ratios were calculated as the ratio of selected AAMRs, including the aggregate male-to-female comparison.

Statistical analysis

Temporal trends in AAMRs were evaluated using the National Cancer Institute Joinpoint Regression Program (version 5.2.0), with calendar year as the independent variable and the natural logarithm of the AAMR as the dependent variable [18]. Annual rate estimates and their standard errors were used in the trend analysis. Models for the overall population, six race categories, and two Hispanic-origin categories were prespecified with 0 joinpoints, yielding one log-linear segment over the six annual observations from 2018 to 2023. No change-point selection or inference about pandemic-specific inflection points was undertaken. Annual percent change (APC) was calculated as 100 × (exp(beta) - 1), where beta is the fitted annual slope. The reported 95% confidence intervals and p-values describe the APC estimates and the null hypothesis of zero temporal change; the nominal two-sided significance threshold was 0.05. Monte Carlo permutation testing for the selection of the number of joinpoints should not be confused with inference for a slope in a model fixed at 0 joinpoints. APC was the primary trend measure; AAPC was not separately reported because there was only one segment. Sex, age, and race-by-sex comparisons remained descriptive. No formal between-group slope comparison or interaction test was performed, so significance in one group and nonsignificance in another does not establish a difference between their trends. Subgroup p-values are interpreted as nominal, exploratory evidence rather than definitive proof of differential trends.

No additional sex-, age-, or race-by-sex trend models or sensitivity analysis excluding 2020-2021 were performed for this revision. Excluding those two years would leave only four observations (2018, 2019, 2022, and 2023), with a two-year internal gap, limiting a stable assessment of whether trends persist outside the pandemic-associated fluctuations. This does not demonstrate robustness to exclusion of pandemic years.

Cause-of-death misclassification is possible because HF is a common terminal pathway for multiple cardiovascular and noncardiovascular conditions and death-certificate detail varies across certifiers. Restricting the analysis to HF as the underlying cause of death may therefore underestimate the broader contribution of HF to mortality. Race and Hispanic-origin classifications on death certificates may differ from Census-derived population denominators, with potentially greater misclassification in some smaller racial groups. The ecological design also precludes individual-level inference because CDC WONDER does not provide patient-level clinical, treatment, insurance, or socioeconomic information. Finally, suppression and unreliability rules may reduce precision in smaller subgroups, particularly in finely stratified analyses.

CDC WONDER suppresses death counts of fewer than 10 to protect confidentiality. Rates flagged by CDC WONDER as unreliable were not treated as valid numeric estimates. Under current NCHS presentation standards [19], rates based on fewer than 10 deaths are suppressed, and estimates based on 10 or more deaths may also be withheld when the relative width of the appropriate 95% confidence interval exceeds 160%; analogous criteria apply to age-adjusted rates using the Fay-Feuer confidence interval. Suppressed or unreliable cells were excluded from rate calculations and were not imputed.

This study used publicly available, de-identified, aggregate mortality data and did not involve identifiable individual patient information. The analysis therefore did not constitute human-subject research requiring institutional review board oversight under the usual interpretation of US federal regulations governing research with publicly available de-identified data.

All data used in this study are publicly available through CDC WONDER [14].

## Results

Baseline burden and demographic differences (2018-2023)

Between 2018 and 2023, 512,960 deaths were attributed to HF as the underlying cause among US adults aged 45 years or older. The aggregate AAMR was 59.6 deaths per 100,000 population. Women accounted for more deaths in absolute terms than men (271,966 versus 240,994), whereas men had a higher aggregate AAMR (68.1 versus 53.0 per 100,000; rate ratio: 1.28) (Table 1).

**Table 1:** Deaths and mortality rates with heart failure as the underlying cause among adults aged 45 years or older (2018-2023) Note: Counts include only deaths with ICD-10 I50 as the underlying cause; they do not represent all deaths involving HF. Rates for sex, race, and Hispanic-origin groups are AAMRs standardized to the US 2000 standard population for the selected age range. *Age-group rows are age-specific crude rates, not AAMRs. All rates are per 100,000 population. Race and Hispanic origin were analyzed separately and may overlap. † N/A: no population denominator was available for Hispanic origin Not Stated. AAMR: age-adjusted mortality rate, HF: heart failure, ICD-10: International Classification of Diseases, 10th Revision

Category	Variable	Deaths (number)	Mortality rate (per 100,000)*
Sex	Female	271,966	53.0
	Male	240,994	68.1
Age group	45-54 years	10,964	4.5*
	55-64 years	32,859	12.9*
	65-74 years	71,321	36.3*
	75-84 years	131,289	131.4*
	85+ years	266,527	692.9*
Race	American Indian or Alaska Native	1,931	29.4
	Asian	9,524	22.9
	Black or African American	59,043	70.9
	Native Hawaiian or Other Pacific Islander	515	41.1
	White	440,288	60.7
	More than one race	1,659	19.8
Ethnicity	Hispanic or Latino	26,827	35.2
	Not Hispanic or Latino	485,375	61.9
	Not Stated	758	N/A†

Marked differences were observed across racial groups. Black or African American adults had the highest aggregate AAMR (70.9 per 100,000; 59,043 deaths). White adults accounted for the largest number of deaths (440,288) and had an AAMR of 60.7 per 100,000. Asian adults had a lower aggregate AAMR than White and Black adults (22.9 per 100,000); the more-than-one-race category had a lower AAMR still (19.8 per 100,000) (Table 1). These population-level differences describe mortality patterns and do not identify their underlying causal mechanisms.

Race-by-sex cross-classification showed that Black men had the highest AAMR among the reported race-sex strata (84.5 per 100,000; 28,304 deaths), compared with 61.2 per 100,000 among Black women (30,739 deaths). White men had an AAMR of 69.0 per 100,000 (206,130 deaths), compared with 54.1 per 100,000 among White women (234,158 deaths). These comparisons were descriptive and were not analyzed as a formal interaction test.

Within the I50 category, most HF deaths were coded as congestive heart failure (I50.0: n = 377,718; 73.6% of deaths with HF as the underlying cause; AAMR = 43.9 per 100,000), followed by heart failure, unspecified (I50.9: n = 132,356; 25.8%; AAMR = 15.4 per 100,000). Left ventricular failure (I50.1) accounted for a small proportion (n = 2,886; 0.6%; AAMR = 0.4 per 100,000).

National temporal pattern

Absolute HF deaths increased from 82,893 in 2018 to 88,830 in 2023. The national AAMR showed year-to-year fluctuation rather than a monotonic increase, including a decrease from 59.5 per 100,000 in 2019 to 58.3 in 2020, and a subsequent increase to 61.0 in 2023 (Figure 1A). Joinpoint regression showed no statistically significant overall temporal trend across 2018-2023 (APC: 0.59%; 95% CI: -0.37 to 1.57; p = 0.227). Thus, the prespecified hypothesis of a significant national increase was not supported.

**Figure 1:**
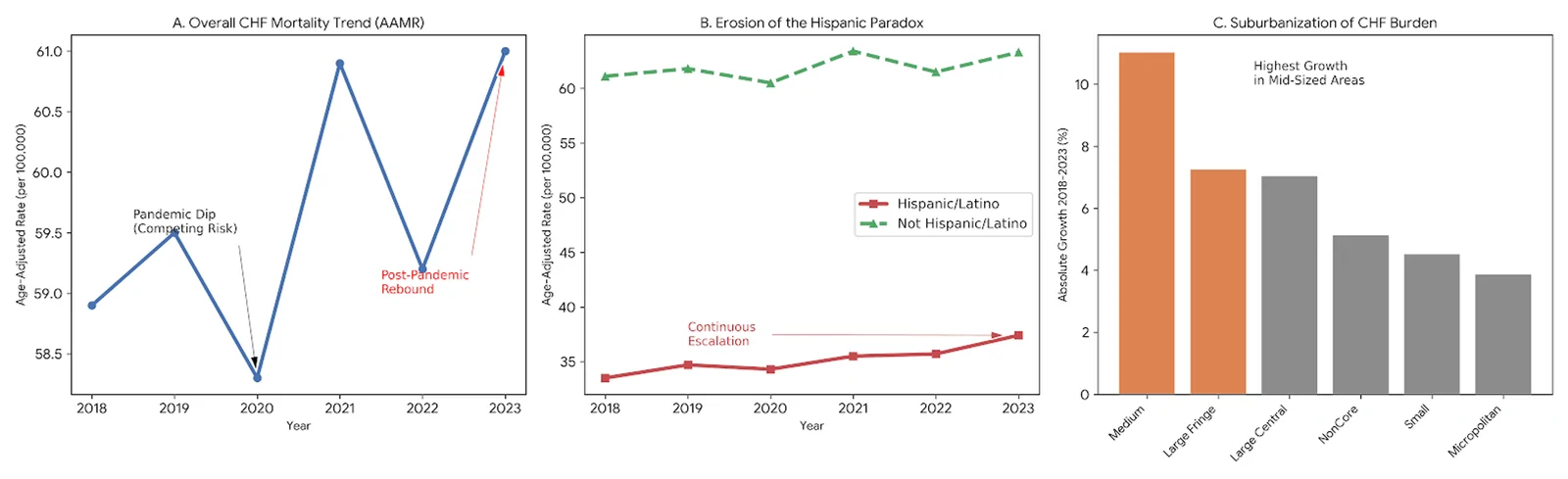
National, Hispanic-origin, and urbanization-stratified patterns in heart failure mortality among US adults aged 45 years or older (2018-2023) (A) Annual overall AAMR, showing year-to-year fluctuation. (B) Annual AAMRs for Hispanic/Latino and Not Hispanic/Latino adults. Hispanic rates remained lower throughout the period but showed an overall upward trend. (C) Percentage change in absolute HF deaths from 2018 to 2023 across NCHS urbanization categories. Panel C depicts absolute burden, not population-adjusted mortality risk. AAMR: age-adjusted mortality rate, CHF: congestive heart failure, HF: heart failure, NCHS: National Center for Health Statistics

Within the largest racial groups, White adults showed no statistically significant temporal trend (APC: 0.83%; 95% CI: -0.20 to 1.86; p = 0.175), and Black or African American adults showed no statistically significant temporal trend (APC: 0.06%; 95% CI: -1.21 to 1.33; p = 0.905) (Table 2).

**Table 2:** Joinpoint trend estimates for underlying-cause heart failure mortality by race and Hispanic origin (2018-2023) Note: * denotes a reported p-value < 0.05 for the null hypothesis of zero APC. Models were prespecified with 0 joinpoints (one log-linear segment) over 2018-2023; no selection among models with different numbers of joinpoints was performed. Estimates, confidence intervals, and p-values are retained from the original analysis. p-values are interpreted at the nominal two-sided 0.05 threshold and are not tests of differences between subgroup slopes. Race includes all Hispanic origins, and Hispanic origin includes all races; the two classifications may overlap. Dashes indicate category headings. APC: annual percent change, CI: confidence interval, HF: heart failure

Population group	Joinpoints	APC	95% confidence interval	p-value
Overall population	0	0.59%	-0.37 to 1.57	0.227
Race	-	-	-	-
American Indian or Alaska Native	0	-0.85%	-9.09 to 8.68	0.744
Asian	0	1.30%*	0.15 to 2.49	0.047
Black or African American	0	0.06%	-1.21 to 1.33	0.905
Native Hawaiian or Other Pacific Islander	0	-2.05%	-5.59 to 1.84	0.277
White	0	0.83%	-0.20 to 1.86	0.175
More than one race	0	-1.77%	-4.69 to 1.55	0.129
Ethnicity	-	-	-	-
Hispanic or Latino	0	1.96%*	0.18 to 3.83	0.030
Not Hispanic or Latino	0	0.61%	-0.45 to 1.70	0.254

Trends by race and Hispanic origin

Race and Hispanic origin were analyzed separately and may overlap. Within the race analysis, Asian adults had an aggregate AAMR of 22.9 per 100,000 and an increasing fitted log-linear trend at the nominal 0.05 significance level (APC: 1.30%; 95% CI: 0.15 to 2.49; p = 0.047) (Figure 2). Annual observed rates fluctuated, so the fitted positive slope should not be interpreted as an increase in every year.

**Figure 2:**
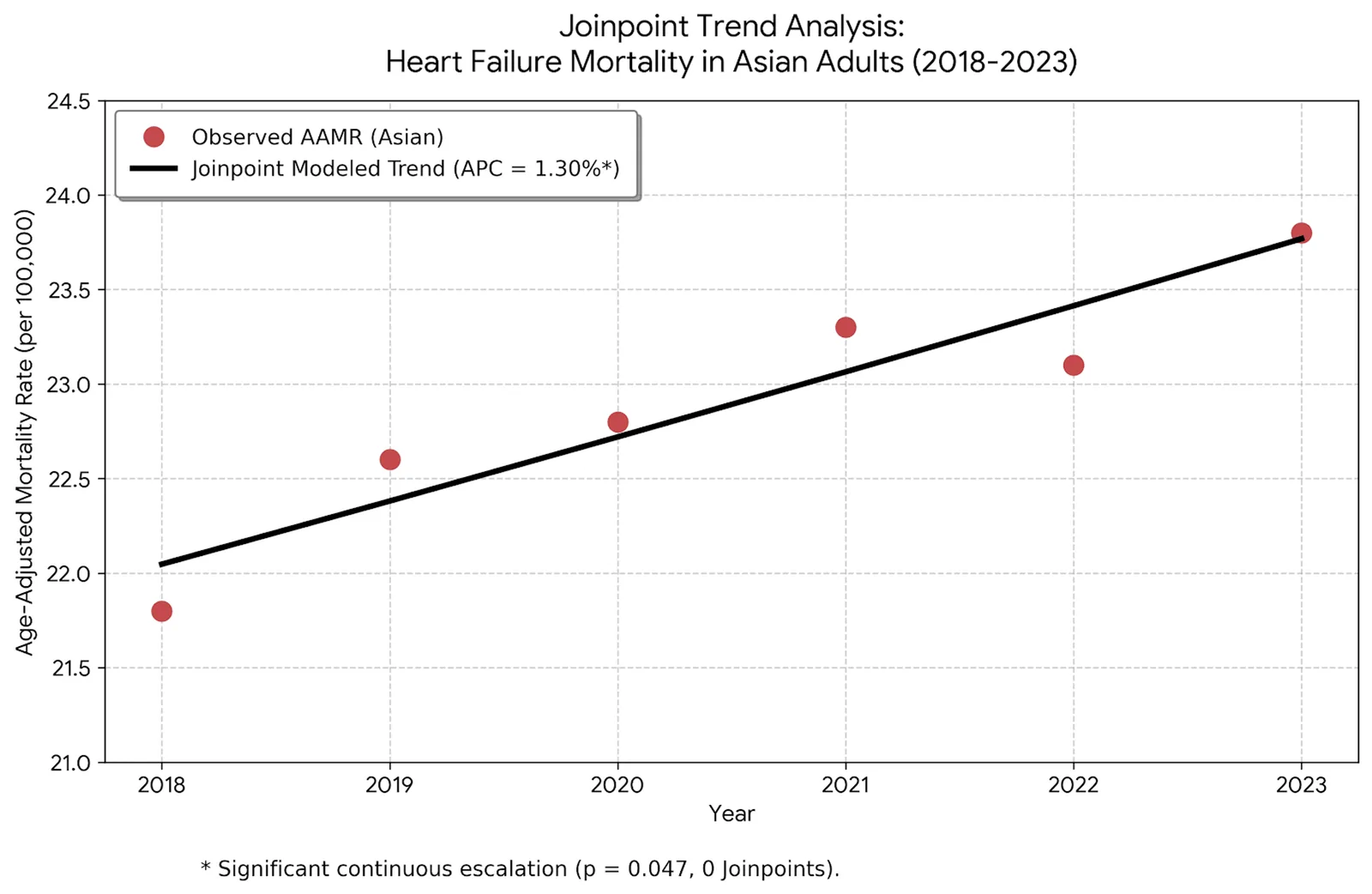
Joinpoint trend analysis of heart failure mortality among Asian adults (2018-2023) Red points show observed annual AAMRs; the solid line shows the fitted log-linear trend. The prespecified model contained 0 joinpoints and yielded an APC of 1.30% (95% CI: 0.15 to 2.49; p = 0.047), a positive slope at the nominal two-sided 0.05 threshold. This within-group test does not compare slopes between population groups. AAMR: age-adjusted mortality rate, APC: annual percent change, CI: confidence interval

Similarly, Hispanic or Latino adults had a substantially lower aggregate AAMR than Not Hispanic or Latino adults (35.2 versus 61.9 per 100,000). Across annual estimates, the Hispanic AAMR increased from 33.5 per 100,000 in 2018 to 37.4 in 2023 (Table 3). Joinpoint regression identified a statistically significant increasing trend among Hispanic adults (APC: 1.96%; 95% CI: 0.18 to 3.83; p = 0.030) (Figure 1B and Figure 3), whereas the trend among Not Hispanic or Latino adults was not statistically significant (APC: 0.61%; 95% CI: -0.45 to 1.70; p = 0.254). These are separate within-group trend estimates. No formal comparison of slopes, interaction analysis, or trend analysis of between-group rate ratios was performed; consequently, these findings do not establish narrowing mortality differences. The ecological design cannot identify the mechanisms underlying the observed patterns.

**Table 3:** Temporal evolution of HF age-adjusted mortality rates* (2018-2023) Note: Values are AAMRs per 100,000 population with 95% confidence intervals, except age-group values, which are age-specific crude rates. Annual changes should be interpreted descriptively; the present ecological analysis did not directly measure competing mortality or establish a causal mechanism for the 2020 changes. Dashes (-) indicate category headers where annual rates are not applicable. AAMR: age-adjusted mortality rate, CI: confidence interval, HF: heart failure

Category/variable	2018	2019	2020	2021	2022	2023
Age group*	-	-	-	-	-	-
45-54 years	3.8 (3.6-4.0)	3.9 (3.7-4.1)	4.7 (4.5-4.9)	5.0 (4.8-5.2)	4.9 (4.6-5.1)	4.7 (4.5-4.9)
55-64 years	11.5 (11.1-11.8)	12.2 (11.9-12.5)	13.1 (12.8-13.4)	13.5 (13.1-13.8)	13.8 (13.5-14.2)	13.6 (13.2-13.9)
65-74 years	34.4 (33.8-35.1)	35.6 (34.9-36.2)	36.5 (35.8-37.2)	37.0 (36.3-37.6)	37.3 (36.6-37.9)	36.6 (36.0-37.2)
75-84 years	132.4 (130.6-134.2)	132.8 (131.1-134.6)	129.7 (128.0-131.5)	134.4 (132.6-136.2)	129.8 (128.2-131.5)	129.7 (128.0-131.3)
85+ years	696.4 (690.0-702.7)	699.5 (693.1-705.9)	663.9 (657.7-670.1)	703.2 (696.5-710.0)	675.0 (668.7-681.3)	722.2 (715.5-728.9)
Race	-	-	-	-	-	-
American Indian or Alaska Native	27.0 (23.7-30.6)	31.3 (27.8-35.1)	29.7 (26.4-33.2)	33.2 (29.8-37.0)	29.6 (26.5-32.9)	26.1 (23.2-29.2)
Asian	22.6 (21.4-23.8)	21.8 (20.7-23.0)	22.8 (21.7-24.0)	23.3 (22.1-24.5)	23.1 (22.0-24.2)	23.8 (22.8-25.0)
Black or African American	69.4 (67.9-70.8)	70.8 (69.4-72.3)	72.8 (71.4-74.3)	71.3 (69.9-72.8)	71.0 (69.6-72.5)	69.9 (68.6-71.4)
Native Hawaiian or Other Pacific Islander	43.8 (34.1-55.4)	44.8 (35.5-55.9)	41.7 (33.0-52.2)	36.0 (28.0-45.7)	41.6 (33.4-51.4)	40.4 (32.6-49.7)
White	59.8 (59.4-60.2)	60.5 (60.0-60.9)	58.8 (58.4-59.2)	62.2 (61.8-62.7)	60.3 (59.9-60.7)	62.7 (62.3-63.1)
More than one race	21.8 (19.2-24.7)	20.2 (17.8-22.9)	19.0 (16.8-21.6)	19.2 (16.9-21.8)	19.4 (17.2-21.8)	19.5 (17.4-21.9)
Ethnicity	-	-	-	-	-	-
Hispanic or Latino	33.5 (32.4-34.6)	34.7 (33.6-35.7)	34.3 (33.3-35.4)	35.5 (34.4-36.6)	35.7 (34.7-36.7)	37.4 (36.3-38.4)
Not Hispanic or Latino	61.1 (60.7-61.5)	61.8 (61.3-62.2)	60.5 (60.1-60.9)	63.4 (63.0-63.9)	61.5 (61.0-61.9)	63.3 (62.9-63.8)

**Figure 3:**
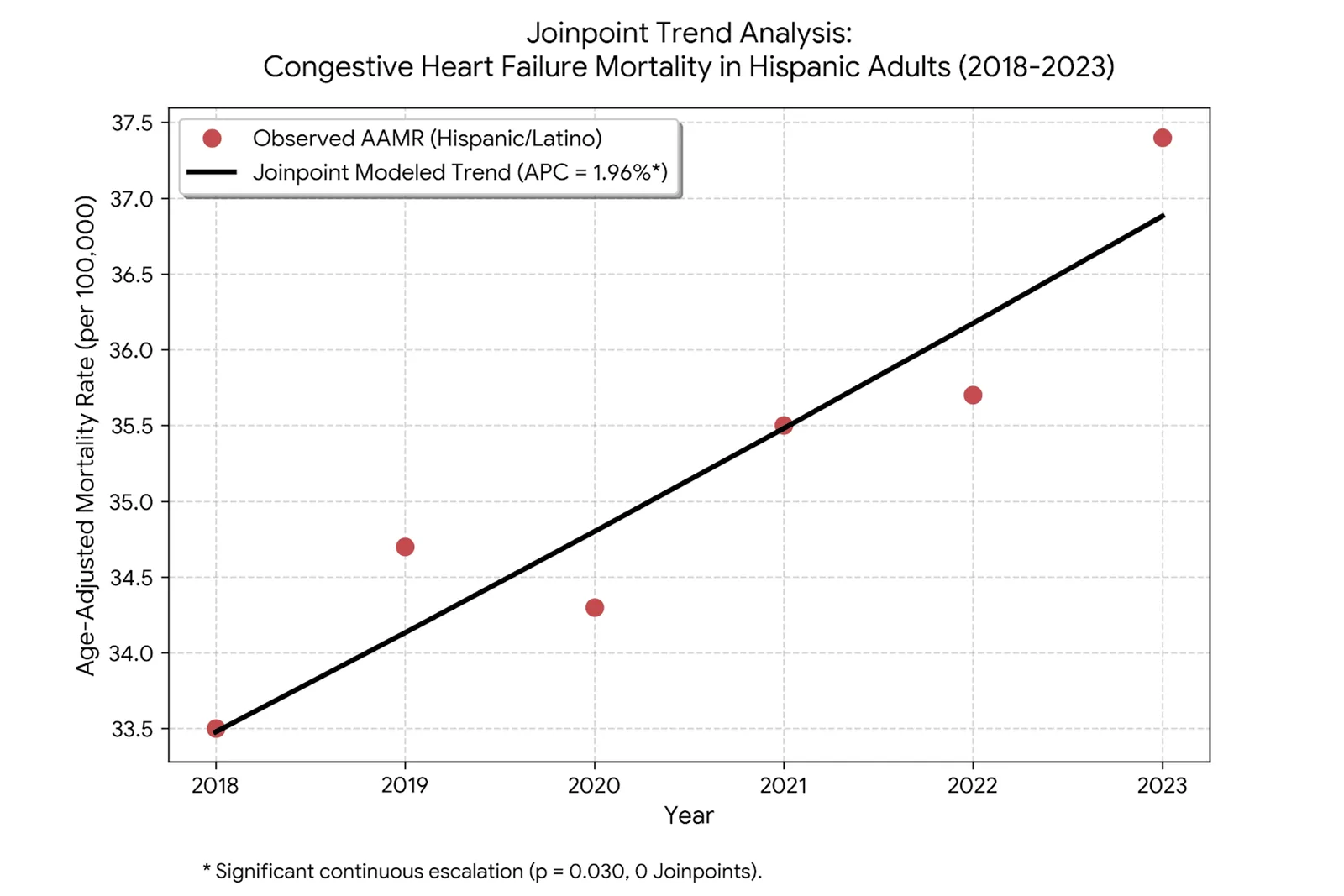
Joinpoint trend analysis of heart failure mortality among Hispanic adults (2018-2023) Red points show observed annual AAMRs; the solid line shows the fitted log-linear trend. The selected model contained 0 joinpoints and yielded an APC of 1.96% (95% CI: 0.18 to 3.83; p = 0.030), indicating a statistically significant increasing trend over the six-year period. AAMR: age-adjusted mortality rate, APC: annual percent change, CI: confidence interval

Urbanization-stratified changes in absolute HF deaths

Across 2018-2023, the largest cumulative numbers of HF deaths occurred in Large Fringe Metro (n = 128,907), Large Central Metro (n = 121,533), and Medium Metro (n = 109,132) counties. Small Metro, Micropolitan, and Noncore counties accounted for 54,078, 53,873, and 45,437 deaths, respectively.

From 2018 to 2023, annual HF deaths increased in all six urbanization categories. The largest percentage increase occurred in Medium Metro counties (+11.0%), followed by Large Fringe Metro (+7.3%), Large Central Metro (+7.1%), Noncore (+5.1%), Small Metro (+4.5%), and Micropolitan (+3.9%) counties (Figure 1C). These values represent changes in absolute death counts and should not be interpreted as population-adjusted differences in mortality risk or as evidence of causal geographic redistribution.

## Discussion

In this nationwide analysis of US adults aged 45 years and older, underlying-cause HF mortality remained substantial from 2018 through 2023 but did not show a statistically significant overall temporal trend (APC: 0.59%; 95% CI: -0.37 to 1.57; p = 0.227). The absence of a statistically significant national slope did not imply uniformity across subgroups. Black adults had the highest aggregate racial AAMR, men had higher AAMR than women, and mortality increased sharply with age. In separate analyses of race and Hispanic origin, increasing trends were observed for Asian and Hispanic groups at the nominal 0.05 significance level. These classifications may overlap, and the results do not constitute a comparison of their slopes. Absolute HF deaths also increased in every urbanization category, with the greatest percentage increase in Medium Metro counties. These findings describe demographic rate differences and variation in geographic death counts, while the hypothesized significant national increase was not detected.

The year-to-year fluctuation surrounding 2020 should be interpreted in the context of well-documented pandemic-era disruption of HF care rather than as evidence of a biologically favorable change in HF risk. Shoaib et al. reported a 40%-47% reduction in HF hospitalizations during the first pandemic wave, accompanied by increased HF mortality in the community; HF deaths at home increased by 31%, and deaths in care homes or hospices increased by 28% during the studied period [20]. In the Get With The Guidelines-Heart Failure registry, Keshvani et al. documented major pandemic-era changes in HF care delivery while showing that many in-hospital quality measures remained comparatively preserved among patients who reached the hospital [21]. Using the US National Inpatient Sample, Mishra et al. found that HF admissions fell by 47% during the initial April 2020 surge and subsequently recovered without returning to pre-pandemic levels [22]. At the population level, Janus et al. identified excess cardiovascular mortality during 2020-2021 and a disproportionate excess HF mortality burden among Black compared with White individuals [23]. Wadhera et al. examined cardiovascular deaths during the early US pandemic period [24], while a subsequent Journal of the American College of Cardiology (JACC) state-of-the-art review summarized the broad disruption of cardiovascular prevention, acute care, and chronic disease management caused by COVID-19 [25]. These studies directly support disrupted care and altered healthcare-seeking behavior as plausible contextual mechanisms for pandemic-era HF mortality patterns. However, the present death-certificate analysis did not measure hospital avoidance, healthcare access, SARS-CoV-2 infection, competing COVID-19 mortality, or care setting, and therefore cannot attribute the 2020 decrease in underlying-cause HF AAMR to any one mechanism.

Viewed over a longer horizon, the present six-year pattern is consistent with the broader loss of progress in US HF mortality. Dimala et al. described an initial decline followed by reversal after 2011, leaving little overall improvement across 2000-2020 [6], while Ullah et al. likewise reported increasing HF mortality after approximately 2012 [7]. Glynn et al. showed that HF mortality declined through 2011 and then increased across all US census regions, with the largest subsequent increases in the Midwest and South [26]. Age-specific analyses have demonstrated that this reversal is not confined to older adults: Jain et al. reported increasing HF-related mortality among young adults [27], whereas Siddiqi et al. demonstrated a renewed increase among adults aged 75 years or older after 2012 [5]. The lack of a significant 2018-2023 slope in our analysis therefore should not be interpreted as restoration of the long-term declines observed earlier in the century; rather, it reflects a short contemporary interval with substantial annual fluctuation.

Racial and ethnic patterns were distinct. Black adults had the highest aggregate AAMR (70.9 per 100,000), but no statistically significant temporal trend was detected during 2018-2023. This indicates that a change was not detected within the available precision; it does not demonstrate a truly constant rate. This interpretation is consistent with contemporary HF and cardiovascular literature. Savitz et al. found higher HF hospitalization among Black patients in a large integrated health system, while mortality relationships varied after adjustment, illustrating that population mortality patterns and individual-level clinical outcomes are not interchangeable [28]. Reviews by Lewsey and Breathett [29] and Piña et al. [30] describe persistent inequities in HF incidence, hospitalization, access to evidence-based therapy, and advanced HF care across racial and ethnic groups. In a national analysis of cardiovascular mortality, Kyalwazi et al. found that Black adults continued to experience higher mortality than White adults, particularly at younger ages and across areas characterized by geographic and structural disadvantage [31]. Our finding of a high aggregate HF AAMR among Black adults is therefore concordant with a broader literature showing persistent inequity, while the ecological design precludes assigning the observed difference to biology, treatment, socioeconomic conditions, structural factors, or any single pathway.

In the race analysis, Asian adults had a lower aggregate AAMR than White and Black adults (22.9 per 100,000); in the separate Hispanic-origin analysis, Hispanic adults had a lower AAMR than non-Hispanic adults (35.2 versus 61.9 per 100,000). Their respective reported APCs were positive at the nominal significance threshold (Asian APC: 1.30%, p = 0.047; Hispanic APC: 1.96%, p = 0.030). These results warrant continued surveillance but do not demonstrate different slopes from other groups or a narrowing mortality gap. A significant within-group test and a nonsignificant test in another group are not equivalent to a significant between-group difference. Broad race and Hispanic-origin categories may also obscure substantial within-group heterogeneity [28-31]. Neither protective characteristics nor changes in acculturation, treatment access, or cardiometabolic risk were measured, and no causal explanation is inferred.

Sex and age further differentiated the mortality burden. Women accounted for more HF deaths in absolute terms, whereas men had a 28% higher aggregate AAMR (68.1 versus 53.0 per 100,000). Contemporary reviews emphasize that sex differences in HF reflect differences in phenotype, risk-factor profiles, cardiac structure and physiology, comorbidities, and treatment evidence, with women more frequently developing HF with preserved ejection fraction [32]. The Heart Failure Society of America epidemiology report likewise describes substantial variation in HF prevalence, phenotype, hospitalization, and outcomes by age and sex [33]. The steep age gradient in our data (from 4.5 per 100,000 among adults aged 45-54 years to 692.9 among those aged 85 years or older) is consistent with HF as a syndrome strongly concentrated in older age groups, while the literature on younger adults demonstrates that adverse trends are also present before old age [27,33]. Because formal Joinpoint testing was not performed separately for sex or age strata in this study, these subgroup comparisons should remain descriptive.

Social and structural determinants provide an important framework for interpreting, but not causally explaining, the observed demographic differences. In a systematic review of 59 HF studies, Enard et al. found that social determinants including race and ethnicity, socioeconomic status, education, and health literacy were repeatedly associated with HF hospitalization, readmission, mortality, and other outcomes, although measurement approaches were heterogeneous [34]. Vinter et al. reported associations between lower socioeconomic position and adverse cardiovascular outcomes among patients with newly diagnosed HF [35]. At the US county level, Khan et al. demonstrated that greater social vulnerability was associated with higher premature cardiovascular mortality, including HF mortality, and that vulnerability clustered with rurality and geographic disadvantage [36]. These observations complement the present results and prior evidence linking social conditions to HF outcomes [9,11,12], but CDC WONDER lacks individual measures of income, education, insurance, neighborhood exposures, medication affordability, or healthcare utilization; these factors therefore remain contextual hypotheses rather than measured mediators in our study.

The urbanization findings require especially careful interpretation because the selected Single-Race CDC WONDER queries did not provide valid denominators for age-adjusted urbanization-specific rates. Our results therefore describe absolute burden, not geographic risk. Prior population-adjusted studies consistently demonstrate a rural disadvantage in HF outcomes. Manemann et al. found that rural residence was independently associated with higher mortality but fewer emergency-department visits and hospitalizations among community patients with HF [37]. Loccoh et al. reported higher 30-day mortality among Medicare beneficiaries presenting to rural rather than urban hospitals with HF (adjusted HR: 1.15) [38]. More recently, Mentias et al. showed lower achievement of optimal guideline-directed medical therapy among rural Medicare beneficiaries with newly diagnosed heart failure with reduced ejection fraction (HFrEF) and estimated that treatment differences accounted for part of the excess rural mortality risk [39]. National mortality analyses have likewise documented higher rural than urban mortality from HF and other major cardiovascular conditions [8,26,40]. Accordingly, our finding that the greatest percentage growth in absolute HF deaths occurred in Medium Metro counties does not contradict a rural mortality disadvantage; it addresses a different quantity and may reflect differences in population size, growth, aging, migration, or mortality.

Changes in where patients die provide an additional context for interpreting mortality counts during periods of healthcare disruption. Before the pandemic, Chuzi et al. documented a long-term US shift in HF-related cardiovascular deaths away from medical facilities and toward home and hospice settings [41]. During COVID-19, Shoaib et al. demonstrated that a sharp fall in HF admissions was accompanied by increased community deaths [20]. These findings strengthen the rationale for caution when interpreting changes in underlying-cause mortality without concurrent data on hospitalization and place of death. The present study did not analyze place of death and therefore cannot determine whether its temporal fluctuations reflect redistribution across care settings.

Impact of the COVID-19 pandemic on HF mortality trajectories

At the individual-patient level, COVID-19 may worsen established HF through inflammatory and myocardial injury, endothelial dysfunction and thrombosis, hypoxemia, arrhythmias, and increased cardiopulmonary stress. Persistent symptoms, impaired functional capacity, and interruptions in follow-up or treatment may further complicate recovery [20-22,25]. The 2026 systematic review by Parizad et al. describes adverse longer-term cardiovascular outcomes among patients with pre-existing HF after COVID-19, including HF deterioration and hospital readmission, and discusses persistent inflammation, vascular dysfunction, and autonomic abnormalities [42]. Its heterogeneous evidence provides clinical context; it does not establish that these mechanisms caused the national mortality pattern observed here.

A temporary decline in deaths assigned to HF as the underlying cause can coexist with worse outcomes among patients who have HF. If COVID-19 initiated the fatal sequence, it could be selected as the underlying cause while HF was recorded as a contributing condition; that death would be excluded from an I50 underlying-cause query [14,15]. This possible diagnostic displacement is distinct from a true improvement in HF control. Competing mortality could also alter the population remaining at risk. These are plausible, untested explanations, not findings of this study: we did not analyze multiple-cause death records, COVID-19 status, or individual competing-risk outcomes.

The six annual observations cannot disentangle biological injury, disrupted care, coding changes, and population change. We did not fit pandemic-specific breakpoints or claim that the 2020 decrease and later fluctuation were caused by COVID-19. Excluding 2020-2021 would leave only 2018, 2019, 2022, and 2023; a four-observation model across this gap would provide limited evidence of robustness. A longer time series and a parallel multiple-cause analysis including HF and COVID-19 would better evaluate these hypotheses.

Strengths and limitations

Several limitations should be considered. First, CDC WONDER provides aggregate death-certificate data without individual-level information on comorbidities, HF phenotype, treatment, medication use, insurance status, socioeconomic conditions, healthcare utilization, or SARS-CoV-2 infection, precluding causal inference. Second, mortality ascertainment depends on death certificates and ICD-10 coding and may be affected by misclassification; restricting HF to the underlying cause captures a narrower component of HF-related mortality than analyses using any-listed or contributing causes. Third, race and Hispanic-origin classification on death certificates may differ from Census-derived population denominators, potentially affecting smaller groups. Fourth, suppression and reliability standards reduce precision for sparse strata. Fifth, pandemic-era competing mortality, healthcare disruption, shifts in care setting, and certification practices complicate interpretation of 2020-2021; although these phenomena are documented in external literature [20-25,41], they were not directly measured here. Sixth, formal trend testing was not performed for sex, age, or race-by-sex strata, so no inferential conclusions about temporal change in those strata are supported. Seventh, the short series, multiple subgroup tests, and p-values close to 0.05 warrant cautious, exploratory interpretation; no formal test established narrowing between-group differences, and robustness to excluding 2020-2021 was not evaluated. Finally, the urbanization analysis lacked the population denominators required for age-adjusted risk comparisons, so geographic results are limited to absolute counts and changes in those counts.

These limitations are balanced by several strengths: a large national sample spanning all 50 states and the District of Columbia; a contemporary six-year period covering 2018-2019 before the emergence of COVID-19 and 2020-2023 after its emergence; standardized national vital-statistics data; explicit restriction to HF as the underlying cause of death; evaluation across multiple demographic dimensions; prespecified Joinpoint trend analyses; and independent dual-analyst data extraction with reconciliation against CDC WONDER. The expanded comparison with contemporary population-based, registry, social-determinant, and pandemic-era studies places the present findings within the broader HF epidemiology literature while preserving the distinction between measured mortality patterns and unmeasured causal mechanisms.

## Conclusions

From 2018 through 2023, no statistically significant overall temporal trend was detected in deaths with HF as the underlying cause among US adults aged 45 years or older. Black adults had the highest aggregate racial AAMR, men had a higher AAMR than women, and age-specific mortality increased sharply with age. The Asian race and Hispanic-origin analyses showed nominally significant positive APCs in separate, potentially overlapping population groups. These within-group findings do not establish differences between subgroup slopes, narrowing mortality gaps, or causal mechanisms.

Absolute HF death counts increased across all six NCHS urbanization categories, most notably in Medium Metro counties. These changes quantify burden and cannot establish population-adjusted geographic risk. The underlying-cause definition excludes deaths in which HF is only contributory and does not quantify the total mortality burden among people with HF. Longer observation periods, multiple-cause mortality analyses, harmonized geographic denominators, and individual clinical and social data are needed to evaluate the mechanisms and robustness of the observed patterns.
